# Four dimensional pulmonary flow evaluation in adolescent patients after surgical and percutaneous pulmonary valve implantation

**DOI:** 10.1186/1532-429X-16-S1-O45

**Published:** 2014-01-16

**Authors:** Soha Romeih, Rob J van der Geest, Arno Roest, Anje M Spijkerboer, Barbara J Mulder, Nico A Blom, Maarten Groenink

**Affiliations:** 1 Radiology, AMC, Amsterdam, Netherlands; 2 Cardiology, AMC, Amsterdam, Netherlands; 3 Radiology, LUMC, Leiden, Netherlands; 4 Paediatrics, LUMC, Leiden, Netherlands; 5 Cardiology, TUH, Tanta, Egypt

## Background

In patients with congenital heart defects, restoration of the connection between the right ventricle (RV) and the pulmonary artery (PA) by surgical or percutaneous valved conduit implantation is a common procedure. Flow characteristics in such conduits are complex, therefore the aim of this study was demonstrating the potential application of 4D MRI flow for the comprehensive assessment of pulmonary flow patterns after surgical and percutaneous RV-PA valved conduit implantation.

## Methods

**Figure 1:**
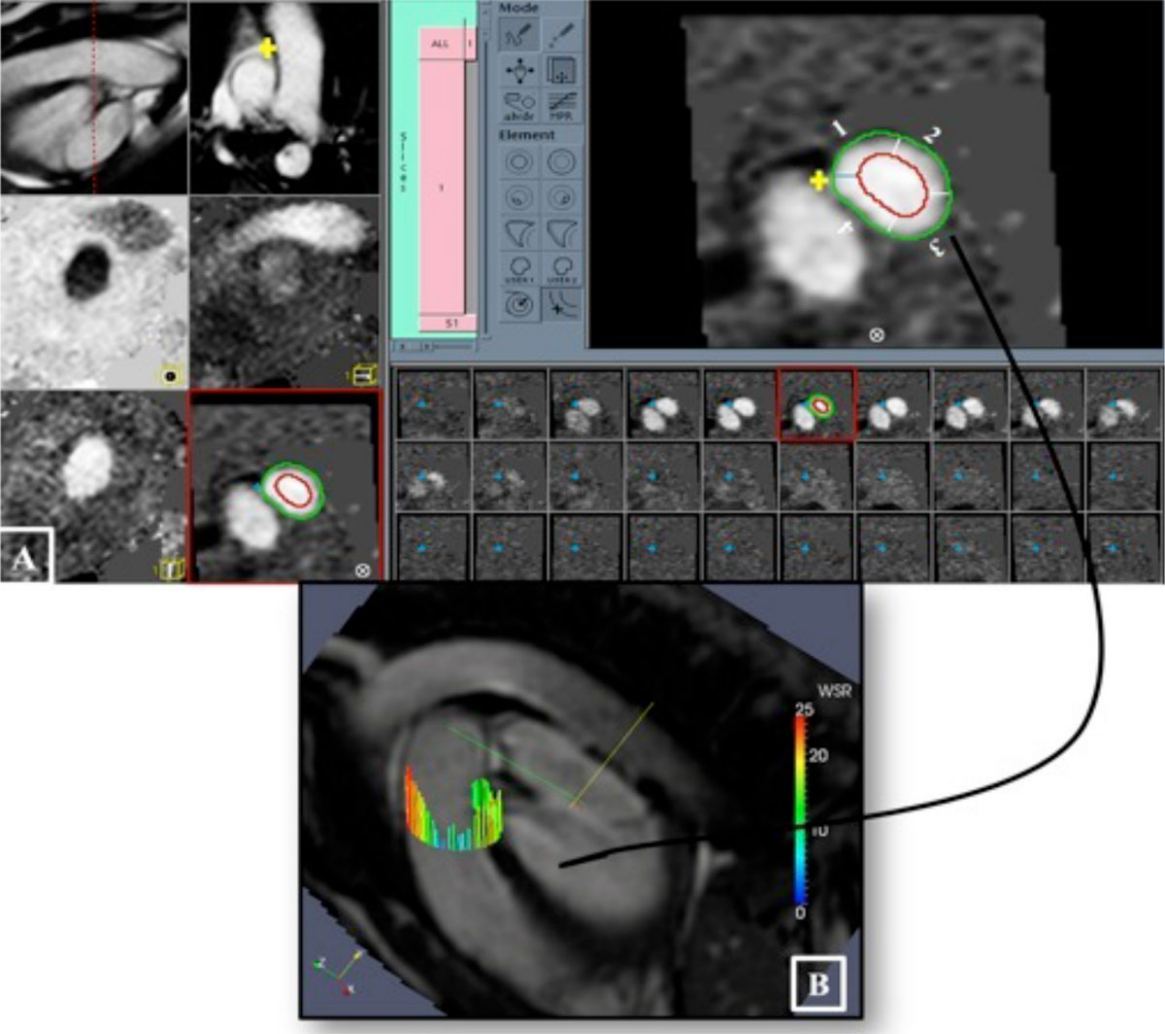
**(A) Quantification of wall shear rates (WSRs) in the phase of maximum velocity (phase 6), the luminal boundary of the pulmonary artery (green contour) was drawn and a concentric contour was generated automatically at a fixed distance of 5 mm inside the lumen (red contour)**. Radial velocity profiles within the region defined by the two contours were analysed to obtain local WSR values. WSRs were calculated at four local anatomical positions starting from the pulmonary wall facing the aorta (the yellow cross, 1), anterior wall (2), lateral wall (3), and posterior wall (4). (B) WSRs measurements

Fifteen patients after surgical RV-PA valved conduit implantation (15.8 ± 1.7 years), 15 patients after percutaneous implantation (17.2 ± 2.0 years), and 15 healthy volunteers (as a control group) (16.5 ± 1.5 years) were included. All subjects underwent a comprehensive cardiac MRI protocol. Analysis focused on evaluation of the RV systolic function and RV mass, the presence of vertical flow patterns, pulmonary flow eccentricity pulmonary artery diameters, and wall shear rate (WSR) assessment. (Figure 1)

## Results

**Figure 2:**
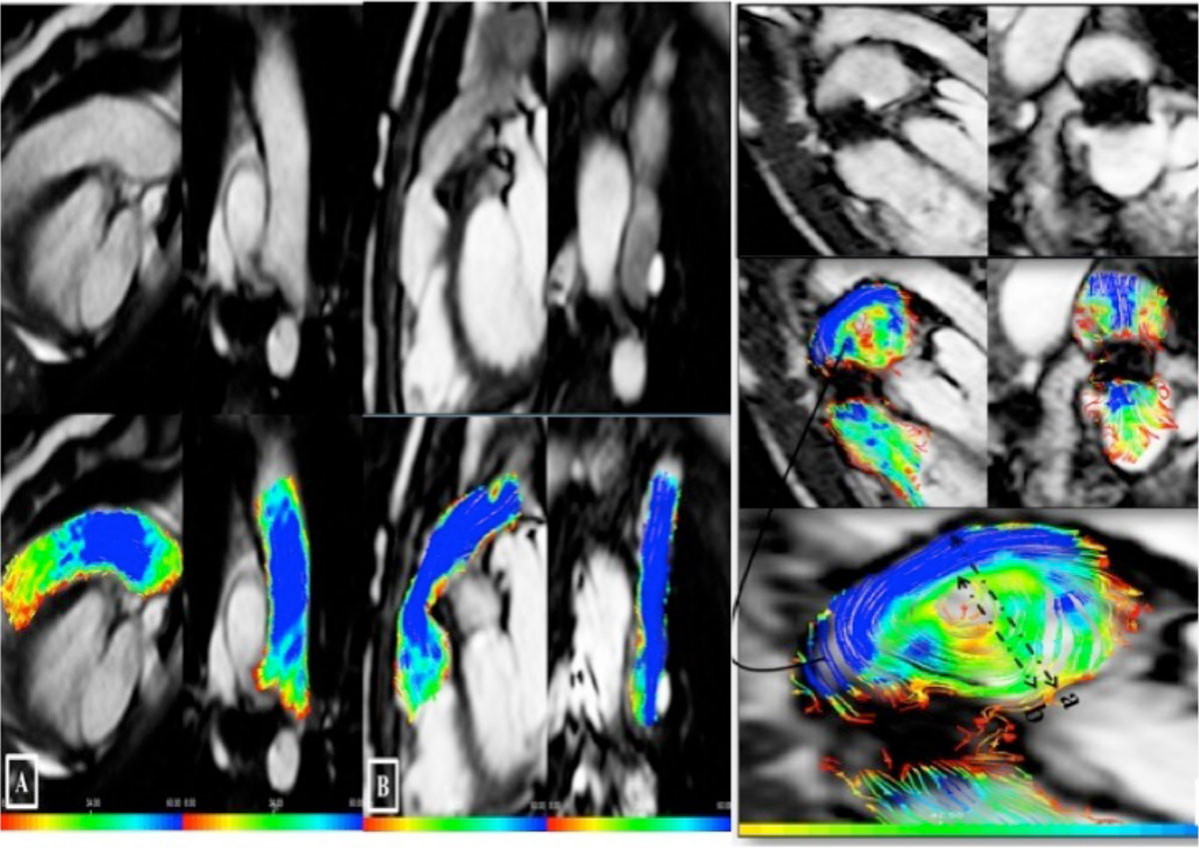
**(A) Pulmonary artery in a normal subject; upper left (sagittal view), and upper right (coronal view)**. (B) Contegra conduit; upper left (sagittal view), upper right (coronal view). Lower rows show traces of color-coded streamlines demonstrating laminar flow, no visible vortex, and no deviation of the flow from the mid line.(C) Melody valve, upper left (sagittal view), upper right (coronal view). Middle row shows traces of color-coded streamlines demonstrating eccentric flow, pulmonary flow is deviated from the midline, and vortex is seen. The lower row is magnification of the vortex in a phase with the maximal vortex diameter. Vortex size measured as a percentage of the vessel diameter: diameter of the vortex (b)/diameter of pulmonary artery (a).

Patients after surgical and percutaneous pulmonary valve implantation showed hypertrophied RV with a good systolic function. Patients with percutaneous implantation showed an eccentric pulmonary flow (deviation angle from the midline 31 ± 10 degree) with vortex appearance (vortex size 73 ± 18%), dilatation of mid RV-PA conduit and a significant asymmetric elevated WSR at focal regions of the conduit. The direction of the jet matched the regions with elevated WSR values. Pulmonary flow deviation angle was positively correlated with the conduit dilatation and with vortex size. In contrast, those after surgical implantation showed a laminar pulmonary flow with no visible vortex and had symmetric, although elevated WSR in the conduit regions. (Figure 2)

## Conclusions

4D MRI flow revealed that pulmonary flow patterns after percutaneous pulmonary valve implantation are markedly different from those after the surgical implantation. Eccentric pulmonary flow is associated with vortex appearance, dilatation of RV-PA conduit, and asymmetrical elevated WSR, which is related to the direction of pulmonary flow jet. This could place this group at risk for future adverse events. Longer follow up studies are required to determine the implications of such knowledge for prognosis and therapy.

## Funding

None.

